# An Innovative Approach to Assess Medical Student Perceived Compassionate Communication Skills Before and After High Acuity Simulation Cases

**DOI:** 10.1177/23821205251408652

**Published:** 2026-01-13

**Authors:** Rachna Subramony, Sophia Aguirre, Grace Furnari, Sandeep Segar, Frances Rudolf

**Affiliations:** 1 Department of Emergency Medicine, 8784 UC San Diego, La Jolla, CA, USA; 2 6684 OHSU, Portland, OR, USA; 3 Department of Medicine, 8784 UC San Diego, La Jolla, Ca, USA

**Keywords:** compassionate communication, empathy, simulation, medical student education, patient–physician relationship

## Abstract

**Objective:**

It can be difficult to translate learned compassionate communication skills in a patient encounter while also managing a high acuity patient. We created a novel curriculum that assesses compassionate communication during challenging high acuity medical simulations for medical students.

**Methods:**

This study was conducted in March 2025 at a large academic medical center during the 4-week Residency Transition Course for all 4th-year medical students (*n* = 120). Participants completed the Sinclair Compassion Questionnaire—Healthcare Professional Ability Self-Assessment (SCQ-HCPASA) prior to simulation and the Sinclair Compassion Questionnaire—Trainee Self-Assessment (SCQ-TSA) immediately after participation. Each student engaged in 5 high-fidelity simulations combining acute medical management with communication challenges involving standardized patients and family members. Data was analyzed descriptively to compare pre- and post-simulation self-perceptions of compassionate communication.

**Results:**

Across all 15 SCQ domains, post-simulation self-assessments demonstrated a mean 18.5-percentage-point decrease in students rating themselves as “often” or “always” able to demonstrate compassionate communication. During debriefs, students shared that their prior education in compassionate communication was limited and primarily classroom-based. They felt confident in their abilities when surveyed initially, however when required to use these skills in real-time while also managing critically ill patients, they found the experience challenging. Students expressed a strong desire for more opportunities to practice compassionate communication in a high-pressure environment, as they found it significantly more challenging than anticipated.

**Conclusion:**

Embedding compassion training within high-acuity simulation identified unrecognized gaps in students’ self-perceived communication abilities. Findings support integrating longitudinal, high-fidelity compassionate communication training earlier in the medical curriculum to better prepare learners for emotionally and cognitively demanding clinical encounters.

## Introduction

Empathy is defined as the ability to recognize and understand the emotions of others. Compassion, however, extends beyond understanding; it compels action, driven by a sincere desire to help, and is rooted in empathy.^1^

Medical education aims to provide students with the essential knowledge and skills for effective medical practice. Among these, empathy and compassion stand out as critical elements in the healthcare setting. In fact, the American Medical Association considers compassion to be integral to the delivery of competent medical care.^2^

Compassionate communication and empathy are teachable skills^3^ that not only correlate with clinical competence,^4^ but also promotes clinician well-being.^5,6^ These skills empower clinicians to address patient and family emotions, disclose medical errors, and deliver difficult news with sensitivity and clarity. Across the United States, medical schools are integrating communication and empathy into their training through simulated case studies, small group discussions, and direct bedside interactions.^3^ Forms of training include formal lectures, videos of patient encounters, interviewing of standardized patients and receiving feedback.^7^ Other programs have promoted reflective writing from the point of view of a hypothetical patient.^8^ Some medical schools have asked their patients to role play clinical situations and even receive saline injections to create empathy for patients who receive intramuscular or subcutaneous medications.^9^ Others have involved mindfulness training with focus on being present and non-judgemental during patient encounters.^10^ Studies have shown that simulation is an effective tool to teach empathy.^11,12^ In one meta-analysis, simulation was found to show improvements in learners’ interpersonal skills and compassionate interactions, ability to establish a comfortable rapport, professionalism, and respect for the patient's physical comfort.^7^ However, many times these simulation experiences are done in an ideal clinical environment that does not take into account barriers such as time constraints and clinical distractions. Proposed interventions to combat these limitations have included doing on the job simulation exercises with residents before a difficult patient encounter, however there is limited data on more closely mimicking the clinical environment during medical student simulations.^13^ Noting the gap in simulation-based curricula designed to teach compassionate communication in the context of acute medical scenarios, we developed a novel simulation based curriculum utilizing standardized patients/actors to play the role of the patient/family members, realistic high acuity clinical scenarios, and a dedicated facilitator to debrief on the communication portion of these simulated sessions.

At our institution, all 4th-year medical students participate in a required 4-week Residency Transition Course (RTC), designed to prepare them for the transition to residency. The RTC serves as the final required course before graduation and is typically held in March, just prior to Match Day. Historically, the simulation sessions within the RTC have focused exclusively on clinical and medical management skills. Students receive empathy and communication training earlier in their education, but these sessions were primarily classroom-based or conducted as one-on-one encounters with standardized patients in controlled, low-stakes environments. In these settings, students were aware in advance that the focus would be communication, which differs significantly from the unpredictability of real-world clinical encounters.

To address this gap, we developed a novel simulation curriculum that integrates high-acuity clinical scenarios with communication challenges. Our goal was to create as realistic a clinical environment as possible through the use of trained standardized patients and actors portraying patients and family members, combined with high acuity medical scenarios. Each simulation was followed by a dedicated debriefing session facilitated by an experienced educator.

Through this curriculum, we aim to better prepare medical students across all specialties for the complex interpersonal demands of modern clinical practice. We hypothesize that by embedding empathy training within high-fidelity, time-pressured simulations, students will identify previously unrecognized gaps in their communication skills. Additionally, our study seeks to identify specific areas for improvement in compassionate communication by administering pre- and post-simulation surveys, assessing perceived changes in empathy and communication performance.

## Materials and Methods

This study was conducted at a large academic medical center with an established simulation-based curriculum for undergraduate medical education in March 2025. All 129 4th-year medical students enrolled in the 2025 RTC were eligible to participate. Nine students were excluded due to excused absences, resulting in a final sample of 120 participants. Students were grouped by their intended residency specialty to enhance engagement and ensure specialty-specific clinical relevance of simulation scenarios.

During the RTC course each student had one, 2-hour simulation session which included 5 high-acuity medical scenarios relevant to each student's intended specialty. For example, patient scenarios for students pursuing internal medicine included a severe COPD exacerbation, sepsis, acute myocardial infarction, altered mental status, and anaphylaxis. During these sessions, either a resident volunteer or faculty course facilitator played the role of a patient's family member. At some point during the case, they would come into the simulation room and ask questions of the team, such as “is my father going to be ok?” or “why does he need to stay in the hospital?,” requiring students to practice simultaneously medically managing a high acuity patient while balancing compassionate communication and limiting use of medical jargon with patients and their families. Students were not informed ahead of time that there would be a family member coming into the room during their medical simulation. During additional cases the students would be interviewing an initially stable patient as a team when the patient would decompensate and require CPR. This case required students to quickly work together to identify a code leader and practice situational awareness and teamwork skills in a stressful clinical case. A structured debrief was led by faculty trained in compassionate communication after each simulation which focused on both medical and communication performance.

Medical students were assessed on the first day of the course using the Sinclair Compassion Questionnaire-Healthcare Provider Competence Self-Assessment (SCQ-HCPASA) (15 items, 5-point Likert scale), a self-assessment tool developed by Sinclair et al to measure health-care professionals’ perceived ability to provide compassion within their workplace context. This tool has demonstrated acceptable internal consistency and was validated in a health-care professional sample.^14,15^

After the simulation session, students completed the Sinclair Compassion Questionnaire—Trainee Self-Assessment (SCQ-TSA) to evaluate perceived growth of compassionate communication skills (see Supplemental material). Both questionnaires used a validated 5-point Likert scale and were analyzed using descriptive statistics to detect pre-post changes and trends across specialty groups. Qualitative outcomes included observations obtained during the debrief sessions, which were used to elicit students’ reflections on their communication style, emotional response, and strategies for improvement. Open-ended feedback was submitted by the students on the post-survey to explore perceived barriers and successes in demonstrating compassion under pressure with this new curriculum. Feedback was also elicited to guide changes to this curriculum in future RTC courses. Questions included how students felt interacting with patients or family members and whether they found it more challenging to communicate with compassion during a high acuity clinical case.

Descriptive statistics were used to summarize responses to each item on the Sinclair Compassion Questionnaire, the Healthcare Professional Ability Self-Assessment (SCQ-HCPASA) and the Sinclair Compassion Questionnaire Trainee Self-Assessment (SCQ-TSA). Frequencies and proportions were calculated for each Likert-scale category to describe pre- and post-simulation perceptions of compassionate communication ability.

For comparison across time points, responses were collapsed into 4 ordinal categories (“Rarely/Lacking,” “Sometimes/Neutral,” “Often/Competent,” and “Always/Very”) to facilitate interpretation. Changes between pre- and post-intervention distributions were examined by calculating percentage-point differences for each item.

Given the aggregated, de-identified nature of the data and absence of individual paired responses, formal inferential testing was not performed. Instead, descriptive trends were analyzed to identify overall shifts in self-reported compassion scores. Data were summarized using means, medians, and ranges across the 15 items to illustrate changes in perceived compassionate communication ability following the simulation intervention.

All analyses were conducted using Microsoft Excel (Microsoft Corp., Redmond, WA).

The reporting of this study conforms to the EQUATOR SQUIRE-SIM Reporting Guidelines^16^ (Supplemental file 8).

## Results

**Figure 1.**
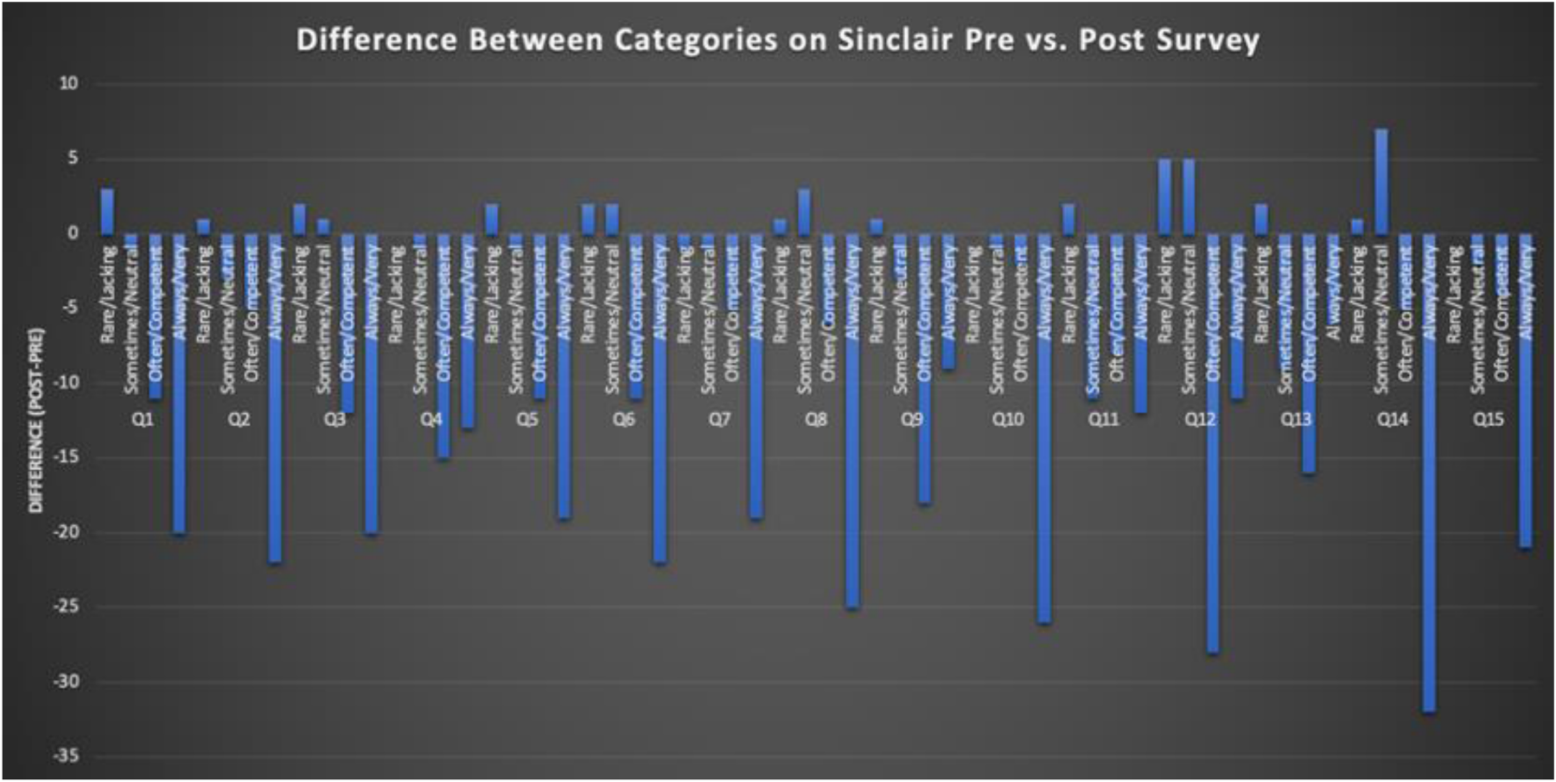
Student responses on the SCQ-TSA compared to the pre-questionnaire SCQ-HCPASA.

**Figure 2.**
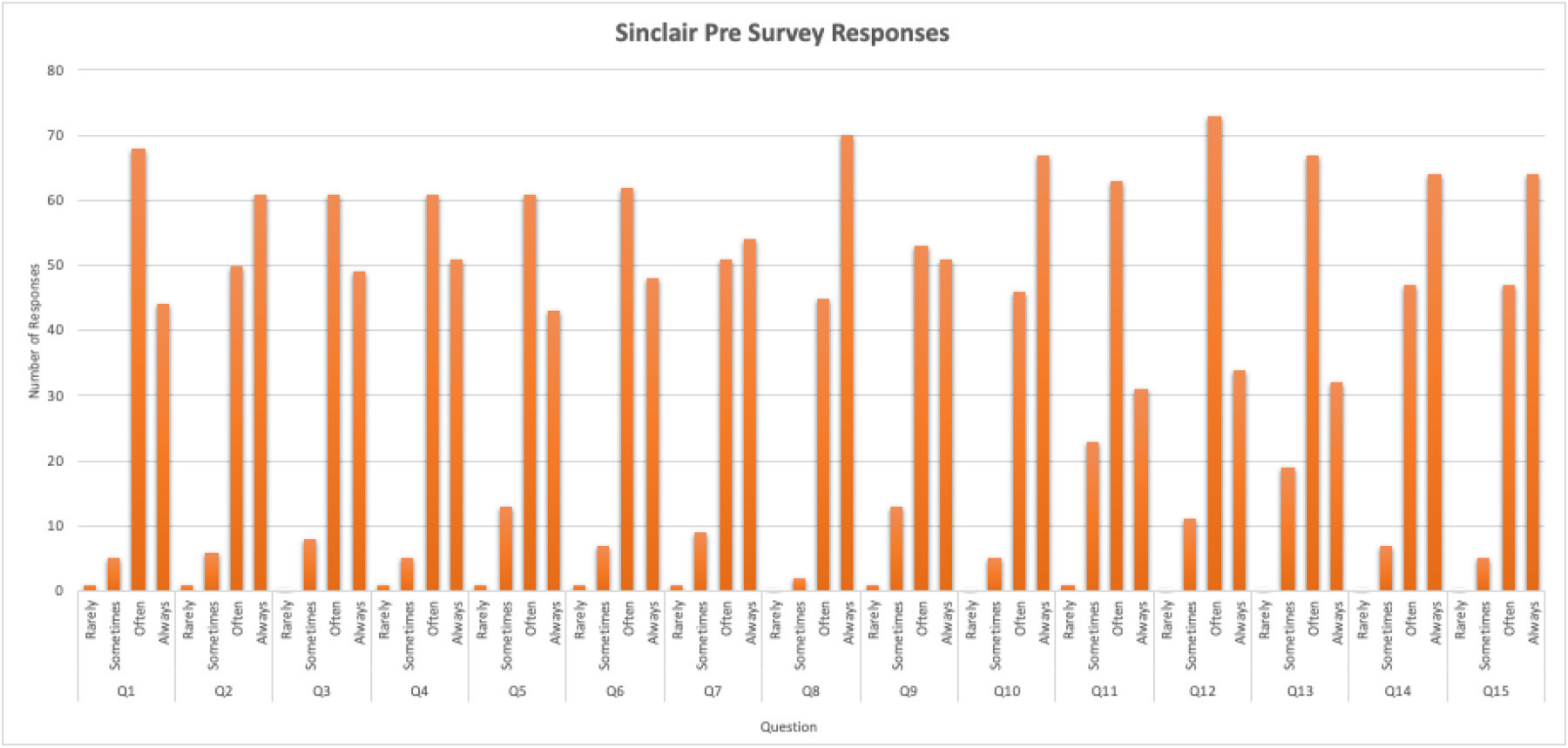
Responses to the Sinclair Compassion Questionnaire—Healthcare Professional Ability Self Assessment (SCQ-HCPASA).

**Figure 3.**
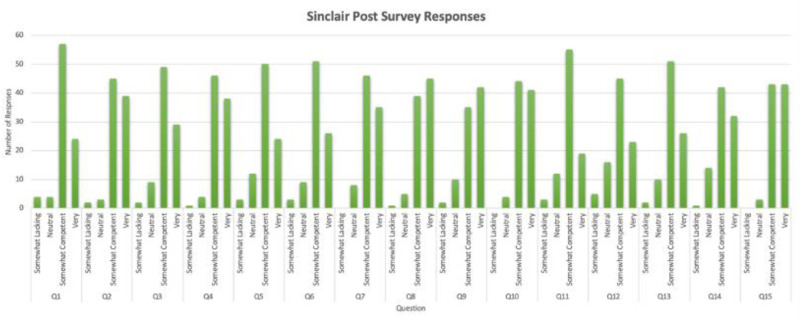
Responses to the the Sinclair Compassion Questionnaire—Trainee Self-Assessment (SCQ-TSA) after completing a series of simulated high acuity patient encounters.

After implementation of this novel high fidelity simulation curriculum, we found that students across all specialties rated their compassionate communication skills at a lower competence level on the SCQ-TSA, the post-simulation exercise assessment survey compared to the initial pre-survey SCQ-HCPASA taken on the first day of the course (Figure 1). In the “rare/lacking” category, the greatest differences were found in question 1 (making the patient feel cared for) and question 12 (forming a good relationship with the patient) with an increase of 3 and 5 respectively compared to the pre-survey (Figures 2 and 3). Question 10 (behaving in a caring way) and question 14 (having a warm presence) had the greatest decrease (−26, −32) in students’ positive ratings of “always/very” for how they showed compassion in these categories (Figures 2 and 3). Quantitative analysis of pre- and post-simulation self-assessments revealed a mean decrease of 18.5 percentage points in the proportion of participants rating themselves in the highest compassion categories. This finding likely represents a recalibration effect commonly observed after experiential learning where participants gain greater insight into the complexity of compassionate communication and thus rate their own ability more conservatively post-training.

Across nearly all questions, students’ rating of their perceived compassion was lower compared to the pre-survey where their compassion ratings were generally higher. Students also responded favorably to this curriculum and debrief sessions with 90% of students rating that they were likely to recommend this program to others. One student also commented that “I think this program uniquely makes sure we addressed or considered the aftermath of a stressful situation. Many times after a case we stop after the diagnosis is done.” Students additionally said that the program highlighted “The importance of connecting with family members and reassuring them even in stressful situations” and that “even in situations of distress/high intensity always remembering the patient is a person.”

## Discussion

Our findings emphasize that students’ perceived compassion is greater when not placed in high acuity situations where they must multitask between medical care and compassionate communication. The students in our study were 4th-year medical students who completed this simulation exercise in the weeks prior to Match Day. All of these medical students had prior training in communication. As these students prepare to enter their intern year, they will need to communicate with patients and family members while also balancing clinical demands in a manner similar to our high fidelity simulation. The results of our study indicate that when faced with more complex scenarios, students felt that their true skills in compassionate communication were inferior to their initial perceptions. Because of this, we advocate for teaching compassionate communication in medical school with this high-fidelity simulation curriculum to better prepare medical students for the real-life clinical environment. The results of our study show an opportunity for a new way of teaching compassionate communication curriculum in medical education. We propose introducing these high fidelity simulations earlier in medical student education, ideally during third-year at the start of clinical clerkships and to expand this curriculum into a 2-year longitudinal course. Given that multiple medical boards are valuing compassion and patient-centered communications by updating exam structures, this is a valuable new initiative in medical school education.

### Limitations

This study has several limitations. First, it used self-reported questionnaires (SCQ-HCPASA and SCQ-TSA) to assess perceived compassionate communication, which may not directly reflect observed behaviors during patient interactions. Self-assessments are subjective and can be influenced by social desirability or recalibration effects, especially following experiential learning activities.

Second, the surveys were administered anonymously and analyzed in aggregate, preventing paired comparison of individual pre- and post-simulation scores. Consequently, only descriptive analyses were performed, and causal inferences regarding the impact of the simulation on compassion development cannot be drawn.

Third, while the SCQ-HCPASA has demonstrated acceptable internal consistency and preliminary validation in healthcare professional samples,^7,14,15^ psychometric testing remains limited outside these populations. Further validation in U.S. medical student cohorts is warranted to confirm its applicability in this context.

Fourth, the study included all 4th-year medical students enrolled in the course rather than a sample determined by a priori power calculation. Although this inclusive approach strengthened representativeness, the absence of a sample size calculation limits the statistical generalizability of the findings.

Fifth, the study was conducted at a single institution within a specific curricular course, which may limit external validity. Replication in multi-institutional settings and across varying educational models is needed to strengthen the generalizability of the results.

Finally, the curriculum primarily focused on compassionate communication. Future iterations should more deliberately integrate non-technical skills such as teamwork, situational awareness, and leadership, competencies that often intersect with compassion and influence communication under pressure.

## Supplemental Material

sj-pdf-1-mde-10.1177_23821205251408652 - Supplemental material for An Innovative Approach to Assess Medical Student Perceived Compassionate Communication Skills Before and After High Acuity Simulation Cases Supplemental material, sj-pdf-1-mde-10.1177_23821205251408652 for An Innovative Approach to Assess Medical Student Perceived Compassionate Communication Skills Before and After High Acuity Simulation Cases by Rachna Subramony, Sophia Aguirre, Grace Furnari, Sandeep Segar and Frances Rudolf in Journal of Medical Education and Curricular Development

sj-docx-2-mde-10.1177_23821205251408652 - Supplemental material for An Innovative Approach to Assess Medical Student Perceived Compassionate Communication Skills Before and After High Acuity Simulation Cases Supplemental material, sj-docx-2-mde-10.1177_23821205251408652 for An Innovative Approach to Assess Medical Student Perceived Compassionate Communication Skills Before and After High Acuity Simulation Cases by Rachna Subramony, Sophia Aguirre, Grace Furnari, Sandeep Segar and Frances Rudolf in Journal of Medical Education and Curricular Development

sj-pdf-3-mde-10.1177_23821205251408652 - Supplemental material for An Innovative Approach to Assess Medical Student Perceived Compassionate Communication Skills Before and After High Acuity Simulation Cases Supplemental material, sj-pdf-3-mde-10.1177_23821205251408652 for An Innovative Approach to Assess Medical Student Perceived Compassionate Communication Skills Before and After High Acuity Simulation Cases by Rachna Subramony, Sophia Aguirre, Grace Furnari, Sandeep Segar and Frances Rudolf in Journal of Medical Education and Curricular Development

sj-pdf-4-mde-10.1177_23821205251408652 - Supplemental material for An Innovative Approach to Assess Medical Student Perceived Compassionate Communication Skills Before and After High Acuity Simulation Cases Supplemental material, sj-pdf-4-mde-10.1177_23821205251408652 for An Innovative Approach to Assess Medical Student Perceived Compassionate Communication Skills Before and After High Acuity Simulation Cases by Rachna Subramony, Sophia Aguirre, Grace Furnari, Sandeep Segar and Frances Rudolf in Journal of Medical Education and Curricular Development
