# Supplementary material for: An Innovative Approach to Assess Medical Student Perceived Compassionate Communication Skills Before and After High Acuity Simulation Cases
Source: J Med Educ Curric Dev. 2026 Jan 13;13:23821205251408652. doi: 10.1177/23821205251408652 (PMC12800009; doi:10.1177/23821205251408652)
Supplement: sj-pdf-1-mde-10.1177_23821205251408652 - Supplemental material for An Innovative Approach to Assess Medical Student Perceived Compassionate Communication Skills Before and After High Acuity Simulation Cases [file sj-pdf-1-mde-10.1177_23821205251408652.pdf]

#810884 - Assessing baseline wellbeing and empathy levels among students at the completion of medical school based on chosen specialty and implementing a novel approach to assess compassionate communication and empathy skills during high acuity specialty specific simulation cases

Protocol Information

Review Type	Status	Approval Date	Continuing Review Date
**Exempt**	**Exempt**	**Jul 11, 2024**	--
Expiration Date	Initial Approval Date	Initial Review Type	
--	**Jul 11, 2024**	**Exempt**	

Feedback

Approval Comment

The above-referenced project has been reviewed by the Director of the UCSD OIA, IRB Chair, or IRB Chair’s designee and is exempt from Institutional Review Board requirements under category(ies) 45 CFR 46.104(d):
(1) Research, conducted in established or commonly accepted educational settings, that specifically involves normal educational practices that are not likely to adversely impact students’ opportunity to learn required educational content or the assessment of educators who provide instruction.
(2): Research that only includes interactions involving educational tests (cognitive, diagnostic, aptitude, achievement), survey procedures, interview procedures, or observation of public behavior (including visual or auditory recording) if the following criteria is met: (ii) Any disclosure of the human subjects' responses outside the research would not reasonably place the subjects at risk of criminal or civil liability or be damaging to the subjects' financial standing, employability, educational advancement, or reputation.

The application listed the following funding (or potential funding) information: Non federal funding: MedEd Seed Grant by the Sanford Institute of Empathy and Compassion - fund E6724 function 400.

This determination does not expire.

Submit amendments to our office only for those changes that could affect exempt status.

Best wishes for the successful conduct of the protocol.

Project Basics

STUDY TITLE
Assessing baseline wellbeing and empathy levels among students at the completion of medical school based on chosen specialty and implementing a novel approach to assess compassionate communication and empathy skills during high acuity specialty specific simulation cases

PRINCIPAL INVESTIGATOR
SUBRAMONY, RACHNA

Lead Department:
Emergency Medicine

Facesheet Inclusion

General Information

SUBMISSION TYPE
Administrative Determination or Registration

Submission for Administrative Determination or Registration.
Human Subjects Research - Exempt Registration

PI is a PI-eligible UCSD employee
Yes

LOCATION WHERE ACTIVITY(IES) WILL BE PERFORMED
UCSD Facilities or Sites (e.g., school, hospital or clinics, etc.)

LAY LANGUAGE SYNOPSIS OF THE PROPOSED ACTIVITY
Literature demonstrates that empathy and compassion decrease during the 4 years of medical school. Simulation is an effective tool to teach empathy. However, many times these simulation experiences are done in an ideal clinical environment that does not take into account real life barriers such as time constraints and clinical distractions. We hope to create a realistic environment for 4th year medical students in the UCSD Residency Transition Course (RTC) with the use of high fidelity simulation mannequins, standardized patients/actors to play the role of the family members, and a dedicated facilitator to debrief on the communication portion of these simulated sessions. We aim to survey 4th year medical students to assess baseline well-being and empathy at the beginning of the RTC course using the Medical Student Well-Being Index (MSWBI) and the Interpersonal Reactivity Index (IRI) surveys. We will assess if there are baseline differences in scores among students based on their chosen specialty. We will then implement a novel curriculum during this course to assess students’ ability to communicate with compassion and empathy in a medically challenging simulated patient encounter. We will use both qualitative and quantitative measures to assesses their ability to medically manage a simulated patient encounter while also speaking plainly and compassionately to patients and family members. Post implementation, we will survey students on the usefulness of these simulated sessions and readminister the MSWBI and IRI surveys at the end of course. Feedback from the survey tools will help guide areas for improvement and additional material to include in future RTC courses.

Study Personnel

Update the PI line by pressing the Edit Pencil and answer the pop-up questions **Do not list any other personnel here. If needed, add an administrative contact in Permissions.**

Person
SUBRAMONY, RACHNA

Home Unit
Emergency Medicine

Institutional Title
Assistant Professor, Department Of Emergency Medicine

Researcher Role
Principal Investigator

Permissions
Please choose **ONLY ONE** option from the below.
Full Access

Person
Aguirre, Sophia

Home Unit
Emergency Medicine

Institutional Title
Resident Physician, Department of Emergency Medicine

Researcher Role
Co-Investigator

Permissions
Please choose **ONLY ONE** option from the below.
Read-Only

Do any of the personnel listed above have any potential conflict of interest related to the research?
No

Funding

Choose the option that describes the funding for this project.
Funding to UCSD planned , but not yet in Proposal Development, IP or Award stage (Department or Fiscal contact plans to submit Proposal, IP or Award record in the Grants Module)

Type of Funding
Non-Federal

Non Federal
Other Non-Federal

For funding that is not or will not be in the UCSD Grants Module, briefly describe type of funding.
MedEd Seed Grant by the Sanford Institute of Empathy and Compassion - fund E6724 function 400

Exempt Basics

I am requesting that UCSD concur with an exemption issued for this project by another institution (for example, the prime awardee).
No

This study (check all that apply)
Recruitment of UCSD/RCHSD faculty, staff, or students

Exempt Categories

All study procedures must fit into one or more of the categories below. Select the applicable category(ies):

1. Educational/Instructional research in an educational setting
Category 1

Each of these statements must be true for your study to qualify for this category:
- Will be conducted in an established or commonly accepted educational setting
- Involves normal educational practices (including research on regular and special education instructional strategies, and research on the effectiveness of or the comparison among instructional techniques, curricula, or classroom management methods)
- Is not likely to adversely impact students’ opportunity to learn required educational content
- Is not likely to adversely impact assessment of educators who provide instruction

My proposed research meets all above criteria. The protocol I will attach provides sufficient detail about how my research meets these criteria.

2. Interactions involving only one or more of the following:
- Educational tests (cognitive, diagnostic, aptitude, achievement)
- Survey procedures with adults (children can be included unless otherwise noted above)
- Interview procedures with adults (children can be included unless otherwise noted above)
- Observation of public behavior (includes visual or auditory recording)

3. Benign behavioral interventions in adults

4. Secondary use of identifiable private information or identifiable biospecimens

5. About public benefit or service programs

6. Taste and food quality evaluation and consumer acceptance studies

Research Characteristics

The research will involve the following. Carefully review then select all that apply or "None of the Above."
Audio, Visual or Digital Recordings
Interview/Focus Groups/Surveys/Questionnaires

This research is cancer-related.
No

Is the use of audio, visual or digital recordings an optional part of the research?
Yes

How will recordings be transcribed?
By the research team (whether manual or automated)

Will study participants be able to review, edit, and erase recordings?
Yes

SCRO Screening

Does this research involve creation or use of a culture-derived, human pluripotent stem cell population derived from an embryo?
No

Does this research involve creation or use of a culture-derived, human pluripotent stem cell population that is a product of somatic cell nuclear transfer (SCNT)?
No

If this project does not include covered Stem Cell Lines then SCRO Review is not required.

Informed Consent

Specify how informed consent will be obtained:
Oral/Implied consent/parental permission

Explain oral/implied consent/parental permission
students will be asked to complete surveys, they will have the opportunity to forgo completion and will not have any bearing on their grade.

Indicate the setting(s) in which the consent process will be conducted:
Group setting

Screening/Recruitment

Does the research require that you conduct a preliminary assessment to determine eligibility?
No

Is this study limited to use of existing identifiable information/data/biospecimens, and will not involve any direct contact with research participants?
No

Specify the recruitment methods to be employed:
Face-to-Face Interaction

Risks & Benefits

Risks to Participants
Does the research involve any physical, psychological, social, legal, economic risks or any other foreseen risks to participants.
No

Do the research activities offer potential direct benefits to research participants?
Yes

Please describe potential direct benefit to the participants.

Note: Payment to participants, whether as an incentive to participate or as compensation for time and inconvenience, is not considered a “benefit.” Also providing incidental care is also not to be considered a benefit.
students will be exposed to a novel curriculum to better supplement their education that aims to help create a realistic clinical environment to prepare them for residency.

Describe the potential societal benefits of the proposed research (e.g., knowledge to be gained):
improved provider communication and empathy. Teaching on better patient care.

Explain how risks to research participants are reasonable in relation to the anticipated benefits:
minimal risk to participants

UCSD/RCHSD Faculty, Staff, Stud.

Will the study include UCSD students?
Yes (specify below)

If yes, does the investigator have grading or supervisory responsibilities for some or all of the students that will be recruited for the study?
Yes (provide justification below)

Enter justification for including own students here...
All UCSD 4th year medical students participate in the RTC course. This is a required course for students to graduate from medical school.

Will the study include UCSD/RCHSD faculty or staff?
Yes (specify below)

If yes, will the investigator be recruiting staff or faculty from his/her own lab or office?
Yes (provide justification below)

Provide justification for including own staff and colleagues here...
Staff currently participates leading simulation sessions and debrief sessions during the RTC course. These staff members will continue to be involved in this course going forward, but will use the new simulation curriculum.

Supporting Information

IRB Certification Required. Upload the following documents, as applicable
- Research Protocol
  - Note: a protocol is not required for studies Exempt under only category 4 (i.e. the only research activity is a secondary use of data)
- Consent/Assent/Parental Permission Forms or Information Sheets, as applicable
- Recruitment Materials, as applicable
- Non-standardized assessments

Supporting Document
yes or no MSWBI.docx
Attachment Type
Data Collection Instruments
Name/Version

Supporting Document
SCQ_Trainee Self-Assessment (SCQ-TSA).pdf
Attachment Type
Data Collection Instruments
Name/Version

Supporting Document
SCQ.pdf
Attachment Type
Data Collection Instruments
Name/Version

Supporting Document
IRI.pdf
Attachment Type
Data Collection Instruments
Name/Version

Supporting Document
UCSD-SBER-Protocol-Template-12.08.21.docx
Attachment Type
Protocol
Name/Version

Supporting Document
Exempt Information Sheet.docx
Attachment Type
Informed Consent/Parental Permission
Name/Version

Supporting Document
citiCompletionCertificate_9994228_60132388.pdf
Attachment Type
Other
Name/Version
CITI PI completion certificate

Added Information - Optional

Include any additional information that you want to communicate about the study.

Assurance/Acknowledgement

By submitting this form, I confirm that the information within this form is accurate and complete.
I am submitting with the awareness and permission of the Principal Investigator

Administrative Details Form

Determinations

Review Type
Exempt

Exemption Cetagory(ies)
Category 1
Category 2(ii)

Study Status
Exempt

Approved Enrollment

Approved at UCSD	Approved at Rady	Approved at All Sites
