# Supplementary material for: An Innovative Approach to Assess Medical Student Perceived Compassionate Communication Skills Before and After High Acuity Simulation Cases
Source: J Med Educ Curric Dev. 2026 Jan 13;13:23821205251408652. doi: 10.1177/23821205251408652 (PMC12800009; doi:10.1177/23821205251408652)
Supplement: sj-docx-2-mde-10.1177_23821205251408652 - Supplemental material for An Innovative Approach to Assess Medical Student Perceived Compassionate Communication Skills Before and After High Acuity Simulation Cases [file sj-docx-2-mde-10.1177_23821205251408652.docx]

| Text Section and Item Notes | SQuIRE 2.0 Items | SQuIRE-SIM Extension |
| --- | --- | --- |
| *Notes to Authors* | - The SQuIRE guidelines provide a framework for reporting new knowledge about how to improve healthcare. - The SQuIRE guidelines are intended for reports that describe system level work to improve the quality, safety, and value of healthcare, and used methods to establish the observed outcomes were due to the intervention(s). - A range of approaches exists for improving healthcare, SQuIRE may be adapted for reporting any of these. - Authors should consider every SQuIRE item, but it may be inappropriate or unnecessary to include every SQuIRE element in a particular manuscript. - The SQuIRE glossary contains definitions of many of the key words in SQuIRE. - The Explanation and Elaboration documents provides specific examples of well-written SQuIRE items, and an in-depth explanation of each item. - Please cite SQuIRE when it is used to write a manuscript. | - The SQuIRE-SIM extension focuses on strategies for reporting simulation-based studies intended to improve healthcare education and/or delivery where simulation is used as an intervention (simulation used to make change) or method of study (simulation to evaluate a change). - For those studies in which simulation is used as an educational intervention, aspects of SQuIRE-EDU may also be applicable. - Authors should consider every SQuIRE and SQuIRE-SIM item, but it may be unnecessary to include every SQuIRE or SQuIRE-SIM element in a particular manuscript. Emphasize "simulation as an intervention" in the intervention sections (#8 & #9), and "simulation as the method of study" in measures (#10) and analysis (#11). Although some information may be relevant in multiple sections of the manuscript, select the optimal section and avoid repetition. - Please cite SQuIRE-SIM when it is used to write a manuscript. - These guidelines may also be useful as a framework when designing a simulation-based quality improvement project to ensure all suggested elements have been met. |
| *Title and Abstract* |  |  |
| 1. Title | Indicate that the manuscript concerns an initiative to improve the healthcare (broadly defined to include the quality, safety, effectiveness, patient-centeredness, timeliness, cost, efficiency and equity of healthcare. | SIM 1: **An Innovative Approach to Assess Medical Student Perceived Compassionate Communication Skills Before and After High Acuity Simulation Cases** |
| 2. Abstract | a. Provide adequate information to aid in searching and indexing.  b. Summarize all key information from various sections of the text using the abstract format of the intended publication or a structured summary such as: background, local problem, methods, interventions, results, conclusions. | SIM 2a: **Keywords include "simulation"**  SIM 2b: **Abstract summarizes all key information from the text and also identifies how simulation is used as the main intervention of our study.** |
| *Introduction: Why did you start?* |  |  |
| 3. Problem Description | Name and significance of the local problem. | **There is a gap in the literature about the effectiveness of teaching high fidelity empathy/compassion training at the medical student level using simulation. We developed a novel curriculum within our University medical school to address this issue.** |
| 4. Available Knowledge | Summary of what is currently known about the problem, including relevant previous studies. | SIM 4: **Literature review conducted including review of meta-analysis on current compassionate communication teaching in medical education. Simulation has been shown to be an effective tool at communication/compassion teaching, however little is known about the effect of high fidelity simulation at the medical school level for compassion training.** |
| 5. Rationale | Informal or formal frameworks, models, concepts and/or theories used to explain the problem, any reasons or assumptions that were used to develop the intervention(s) and reasons why the intervention(s) was expected to work. | SIM 5: **Our rationale for using simulation as an intervention was because it was already being used to train medical students on medical knowledge. We figured that we could easily make the transition to using this to also teach compassionate communication by just making small changes to simulation cases that medical students were already accustomed to doing.** |
| 6. Specific Aims | Purpose of the project and of this report. | **To create new high fidelity simulation curriculum for medical students to teach compassionate communication in a way that mirrors the real life clinical environment.**  **To identify knowledge gaps for students and to better address these gaps with our new curriculum.**  **To elicit feedback on this novel curriculum for future medical student education endeavors and to eventually create a new, more effective way at teaching empathy and compassion in medical education.** |
| *Methods: What did you do?* |  |  |
| 7. Context | Contextual elements considered important at the outset of introducing the intervention(s). | SIM 7: **Our study was conducted in March of 2025 at our University medical school with students participating in the Residency Transition Course during their 4th year of medical school. The Residency Transition Course is a required course for all 4th year medical students who are matching and preparing to start residency. During the RTC course each student had one, two-hour simulation session which included five high-acuity medical scenarios relevant to each student’s intended specialty.** |
| 8. Intervention(s) | a. Description of the intervention(s) in sufficient detail that others could reproduce it.  b. Specifics of the team involved in the work. | 1. **Students were grouped by specialty and rotated through a two hour simulation session at different allotted time slots in their RTC schedule. Each specialty group participated in 5 different medical scenarios and were not told ahead of time that there would be compassion components to each of these clinical scenarios. For example, patient scenarios for students pursuing internal medicine included a severe COPD exacerbation, sepsis, acute myocardial infarction, altered mental status, and anaphylaxis. During these sessions, either a resident volunteer or faculty course facilitator played the role of a patient’s family member. At some point during the case, they would ask questions of the team, such as “is my father going to be ok?” or “why does he need to stay in the hospital?”, requiring students to practice simultaneously medically managing a high acuity patient while balancing compassionate communication and limiting use of medical jargon with patients and their families. Students were not informed ahead of time that there would be a family member coming into the room during their medical simulation.** 2. **Team members included a group of medical students broken up by specialty, a physician facilitator in a separate room manning the mannequin and vital signs, and an additional facilitator or resident volunteer who would play the role of the family member or patient in some cases.** |
| 9. Studies of the Intervention(s) | a. Approach chosen for assessing the impact of the intervention(s).  b. Approach used to establish whether the observed outcomes were due to the intervention. | SIM 9a: **Our intervention was using prior medical cases and adding in compassion aspects to each case to simulate real life situations for the students.**  SIM 9b: **Variability across simulations was limited by having prewritten cases and using the same cases for each simulation day. Variability was also limited among facilitators playing the role of the family member by having these facilitators all use similar scripts and doing a training with them ahead of time.** |
| 10. Measures | a. Measures chosen for studying processes and outcomes of the intervention(s), including rationale for choosing them, their operational definitions and their validity and reliability.  b. Description of the approach to the ongoing assessment of contextual elements that contributed to the success, failure, efficiency and cost.  c. Methods employed for assessing completeness and accuracy of data. | SIM 10a: **Simulation was chosen as an intervention as it is a tool that most closely mimics the clinical environment, which was a goal of our study.**  SIM10b: **Somewhat limited given that students were not actually in a real life scenario. Realism was closely achieved with realistic intern level clinical scenarios, real time vital sign monitoring, high fidelity mannequins with breath sounds and other physical exam findings, and an actor as the family member as opposed to a simulated mannequin or the compassion aspect.**  SIM 10c: **Our simulation center provided real life video recordings of students during these scenarios. Students also completed their own surveys directly after the experience to limit recall bias.** |
| 11. Analysis | a. Qualitative and quantitative methods used to draw inferences from the data  b. Methods for understanding variation within the data, including the effects of time as a variable. | 1. **Student survey results from day one of RTC and immediately after participating in simulation were compared. We also received qualitative written feedback from the students about their experiences and areas of improvement.**   SIM 11b: **Time was a limited variable in our study as immediately after the simulation was completed, students were given time to fill out their surveys and also debrief the scenario.** |
| 12. Ethical Considerations | Ethical aspects of implementing and studying the intervention(s) and how they were addressed, including, but not limited to, formal ethics review and potential conflict(s) of interest. | SIM 12: **Students were informed of the study on day one of the RTC course and responses were kept anonymous. Students were told that they could opt out of the study by not filling out the form and this would have no effect on grading or performance. We were blinded to which students filled out the survey and which students didn’t.** |
| *Results: What did you find:* |  |  |
| 13. Results | a. INitial steps of the intervention(s) and their evolution over time (e.g. time-line diagram, flow chart or table), including modifications made to the intervention during the project.  b. Details of the process measures and outcomes.  c. Contextual elements that interacted with the intervention(s).  d. Observed associations between outcomes, intervention(s) and relevant contextual elements.  e. Unintended consequences such as unexpected benefits, problems, failures or costs associated with the intervention(s).  f. Details about missing data. | **Across nearly all questions, students' rating of their perceived compassion was lower compared to the pre survey where their compassion ratings were generally higher. Students also responded favorably to this curriculum and debrief sessions with 90% of students rating that they were likely to recommend this program to others. One student also commented that “I think this program uniquely makes sure we addressed or considered the aftermath of a stressful situation. Many times after a case we stop after the diagnosis is done.” Students additionally said that the program highlighted “The importance of connecting with family members and reassuring them even in stressful situations” and that “even in situations of distress/high intensity always remembering the patient is a person.” Using a validated survey tool (sinclair compassion question), students were surveyed on their perceived compassionate communication pre and post simulation.**  **In the “rare/lacking” category, the greatest differences were found in question 1 (making the patient feel cared for) and question 12 (forming a good relationship with the patient) with an increase of 3 and 5 respectively compared to the pre survey (Figure 1, 2). Question 10 (behaving in a caring way) and question 14 (having a warm presence) had the greatest decrease (-26, -32) in students’ positive ratings of “always/very” for how they showed compassion in these categories (Figure 1, 2). Quantitative analysis of pre- and post-simulation self-assessments revealed a mean decrease of 18.5 percentage points in the proportion of participants rating themselves in the highest compassion categories. This finding likely represents a recalibration effect commonly observed after experiential learning where participants gain greater insight into the complexity of compassionate communication and thus rate their own ability more conservatively post-training** |
| *Discussion: What does it mean?* |  |  |
| 14. Summary | a. Key findings, including relevance to the rationale and specific aims.  b. Particular strengths of the project. | 1. **The results of our study indicate that when faced with more complex scenarios, students feel that they had difficulty with their compassionate communication skills even though their initial perception of these skills was higher. Because of this, we advocate for teaching compassionate communication in medical school with this high-fidelity simulation curriculum to better prepare medical students for the real life clinical environment. The results of our study show an opportunity for a new way of teaching compassionate communication curriculum in medical education.** 2. **Simulation was a strength in our project design as it allowed for us to create an innovative high fidelity environment for medical students to practice communication.** |
| 15. Interpretation | a. Nature of the association between the intervention(s) and the outcomes.  b. Comparison of results with findings from other publications.  c. Impact of the project on people and systems.  d. Reasons for any differences between observed and anticipated outcomes, including the influence of context.  e. Costs and strategic trade-offs, including opportunity costs. | **Our study produced our desirable outcome as student’s perceptions of their empathy decreased post intervention, indicating to us that there is a need for new high fidelity teaching of empathy and communication to better prepare students for the reality of clinical care. Similar to other studies, our intervention does highlight the importance of simulation in medical student empathy teaching, however our student introduces a novel way of using simulation to teach empathy and communication. This will further impact medical students to come at our institution as it was received favorably. We hope the results of our study will also inspire changes to empathy training in medical school at other institutions.** |
| 16. Limitations | a. Limits to the generalizability of the work.  b. Factors that might have limited internal validity such as confounding, bias or imprecision in the design, methods, measurements or analysis.  c. Efforts made to minimize and adjust for limitations. | **Limitations:**   - **Self reported questionnaire** - **Single site** - **Our survey has been validated for healthcare professionals and not validated for students**   Efforts were made to limit recall bias by having students complete the post survey immediately after the simulation. |
| 17. Conclusions | a. Usefulness of work.  b. Sustainability  c. Potential for spread to other contexts.  d. Implication for practice and for further study in the field.  e. Suggested next steps. | **We believe our study is useful at helping to change the landscape of compassionate communication medical student education. It is sustainable as it requires minimal effort to amend simulation cases to include communication aspects as students at many medical schools are already doing these medical case based simulations as a part of their curriculum. We hope to replicate this intervention in 1-3rd year medical students to help them be better prepared for residency and to also implement this at other institutions.** |
| 18 Funding | Sources of funding that supported this work. Role, if any, of the funding organization in the design, implementation, interpretation and reporting | **Our study was funded by a grant from the Sanford institute that allowed for compensation of facilitators for their time and curriculum maintenance for the RTC course.** |
