# Supplementary material for: An Innovative Approach to Assess Medical Student Perceived Compassionate Communication Skills Before and After High Acuity Simulation Cases
Source: J Med Educ Curric Dev. 2026 Jan 13;13:23821205251408652. doi: 10.1177/23821205251408652 (PMC12800009; doi:10.1177/23821205251408652)
Supplement: sj-pdf-3-mde-10.1177_23821205251408652 - Supplemental material for An Innovative Approach to Assess Medical Student Perceived Compassionate Communication Skills Before and After High Acuity Simulation Cases [file sj-pdf-3-mde-10.1177_23821205251408652.pdf]

The Sinclair Compassion Questionnaire – Healthcare Professional Ability Self Assessment (SCQ-HCPASA)

This questionnaire has been developed to ask you about your experience with the following aspects of compassion. Please carefully read each question and rate how often you feel you can do each of the following.

Instructions:

Please rate how often you are able to do the following in your work environment:

1. Making my patients feel cared for

○ Never able ○ Rarely able ○ Sometimes able ○ Often able ○ Always able

2. Showing genuine concern for my patients

○ Never able ○ Rarely able ○ Sometimes able ○ Often able ○ Always able

3. Communicating with my patients in a sensitive manner

○ Never able ○ Rarely able ○ Sometimes able ○ Often able ○ Always able

4. Being attentive to my patients

○ Never able ○ Rarely able ○ Sometimes able ○ Often able ○ Always able

5. Providing comfort to my patients

○ Never able ○ Rarely able ○ Sometimes able ○ Often able ○ Always able

6. Being very supportive when talking to my patients

○ Never able ○ Rarely able ○ Sometimes able ○ Often able ○ Always able

7. Providing care to my patients in a gentle manner

○ Never able ○ Rarely able ○ Sometimes able ○ Often able ○ Always able

© 2022 UTI Limited Partnership

8. Speaking to my patients with kindness

○ Never able ○ Rarely able ○ Sometimes able ○ Often able ○ Always able

9. Seeing my patients as a person and not just a patient

○ Never able ○ Rarely able ○ Sometimes able ○ Often able ○ Always able

10. Behaving in a caring way when interacting with my patients

○ Never able ○ Rarely able ○ Sometimes able ○ Often able ○ Always able

11. Really understanding my patients' needs

○ Never able ○ Rarely able ○ Sometimes able ○ Often able ○ Always able

12. Establishing a good relationship with my patients

○ Never able ○ Rarely able ○ Sometimes able ○ Often able ○ Always able

13. Seeing things from my patients' perspective

○ Never able ○ Rarely able ○ Sometimes able ○ Often able ○ Always able

14. Conveying a warm presence to my patients

○ Never able ○ Rarely able ○ Sometimes able ○ Often able ○ Always able

15. Being sincere with my patients

○ Never able ○ Rarely able ○ Sometimes able ○ Often able ○ Always able

© 2022 UTI Limited Partnership
