# Supplementary material for: An Innovative Approach to Assess Medical Student Perceived Compassionate Communication Skills Before and After High Acuity Simulation Cases
Source: J Med Educ Curric Dev. 2026 Jan 13;13:23821205251408652. doi: 10.1177/23821205251408652 (PMC12800009; doi:10.1177/23821205251408652)
Supplement: sj-pdf-4-mde-10.1177_23821205251408652 - Supplemental material for An Innovative Approach to Assess Medical Student Perceived Compassionate Communication Skills Before and After High Acuity Simulation Cases [file sj-pdf-4-mde-10.1177_23821205251408652.pdf]

The Sinclair Compassion Questionnaire – Trainee Self-Assessment (SCQ-TSA)

This self-assessment has been developed to assess your clinical competence related to the following aspects of compassionate care. It can be filled out immediately following a clinical visit with actual or standardized patients. Please carefully read each question and rate your level of agreement regarding your competence.

Instructions:
Please rate your competence in the following aspects of compassion:

1. I made the patient feel cared for.

○ Not at all competent ○ Somewhat lacking competence ○ Neutral ○ Somewhat competent ○ Very competent

2. I showed genuine concern for the patient.

○ Not at all competent ○ Somewhat lacking competence ○ Neutral ○ Somewhat competent ○ Very competent

3. I communicated with the patient in a sensitive manner.

○ Not at all competent ○ Somewhat lacking competence ○ Neutral ○ Somewhat competent ○ Very competent

4. I was attentive to the patient.

○ Not at all competent ○ Somewhat lacking competence ○ Neutral ○ Somewhat competent ○ Very competent

© 2020 UTI Limited Partnership

5. I provided comfort to the patient.

○ Not at all competent ○ Somewhat lacking competence ○ Neutral ○ Somewhat competent ○ Very competent

6. I was very supportive when talking to the patient.

○ Not at all competent ○ Somewhat lacking competence ○ Neutral ○ Somewhat competent ○ Very competent

7. I provided care to the patient in a gentle manner.

○ Not at all competent ○ Somewhat lacking competence ○ Neutral ○ Somewhat competent ○ Very competent

8. I spoke to the patient with kindness.

○ Not at all competent ○ Somewhat lacking competence ○ Neutral ○ Somewhat competent ○ Very competent

9. I saw the patient as a person and not just a patient.

○ Not at all competent ○ Somewhat lacking competence ○ Neutral ○ Somewhat competent ○ Very competent

10. I behaved in a caring way when interacting with the patient.

○ Not at all competent ○ Somewhat lacking competence ○ Neutral ○ Somewhat competent ○ Very competent

© 2020 UTI Limited Partnership

Compassion Research Lab

11. I really understood the patient’s needs.

- ○ Not at all competent
- ○ Somewhat lacking competence
- ○ Neutral
- ○ Somewhat competent
- ○ Very competent

12. I established a good relationship with the patient.

- ○ Not at all competent
- ○ Somewhat lacking competence
- ○ Neutral
- ○ Somewhat competent
- ○ Very competent

13. I was able to see things from the patient’s perspective.

- ○ Not at all competent
- ○ Somewhat lacking competence
- ○ Neutral
- ○ Somewhat competent
- ○ Very competent

14. I had a warm presence.

- ○ Not at all competent
- ○ Somewhat lacking competence
- ○ Neutral
- ○ Somewhat competent
- ○ Very competent

15. I was sincere with the patient.

- ○ Not at all competent
- ○ Somewhat lacking competence
- ○ Neutral
- ○ Somewhat competent
- ○ Very competent

© 2020 UTI Limited Partnership
